# Supplementary material for: Self-Standing Pd-Based Nanostructures for Electrocatalytic CO Oxidation: Do Nanocatalyst Shape and Electrolyte pH Matter?
Source: Int J Mol Sci. 2023 Jul 23;24(14):11832. doi: 10.3390/ijms241411832 (PMC10380336; doi:10.3390/ijms241411832)
Supplement: Supplementary file 1 [file ijms-24-11832-s001.zip › ijms-2438056-supplementary.pdf]

Supporting Information

# Self-Standing Pd-Based Nanostructures for Electrocatalytic CO Oxidation: Do Nanocatalyst Shape and Electrolyte pH Matter?

**Belal Salah [1,2,†], Adewale K. Ipadeola [1,2,†], Aboubakr M. Abdullah [1,*], Alaa Ghanem [3] and Kamel Eid [2,*]**

[1] Center for Advanced Materials, Qatar University, Doha 2713, Qatar
[2] Gas Processing Center (GPC), College of Engineering, Qatar University, Doha 2713, Qatar
[3] PVT-Lab, Production Department, Egyptian Petroleum Research Institute, Cairo 11727, Egypt
[*] Correspondence: bakr@qu.edu.qa (A.M.A.); kamel.eid@qu.edu.qa (K.E.)
[†] These authors contributed equally to this work.

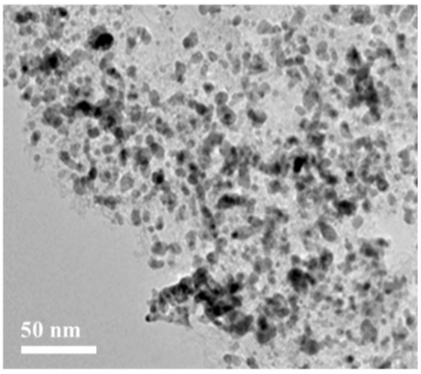


Figure S1. TEM of the Pd/C.

Table S1. Binding energies of Pd 3d in Pd nanocube, Pd nanosponge, and Pd/C catalysts.

| Catalyst | Pd $3d_{5/2}$ | | Pd $3d_{3/2}$ | |
|---|---|---|---|---|
| | $Pd^0$ | $Pd^{2+}$ | $Pd^0$ | $Pd^{2+}$ |
| Pd nanocube | 333.52 | 335.43 | 338.93 | 342.49 |
| Pd nanosponge | 333.73 | 335.62 | 338.94 | 341.34 |

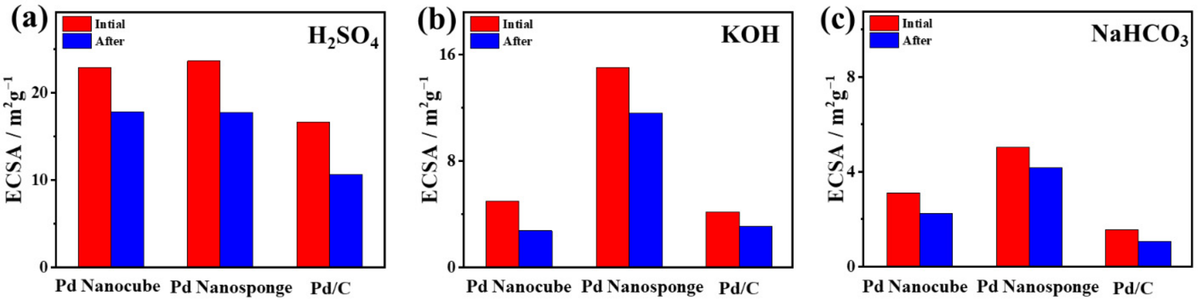


Figure S2. The ECSA before and after electrochemical CO oxidation stability (a) 0.1 M $H_2SO_4$, (b) 0.1 M KOH and (c) 0.5 M $NaHCO_3$.

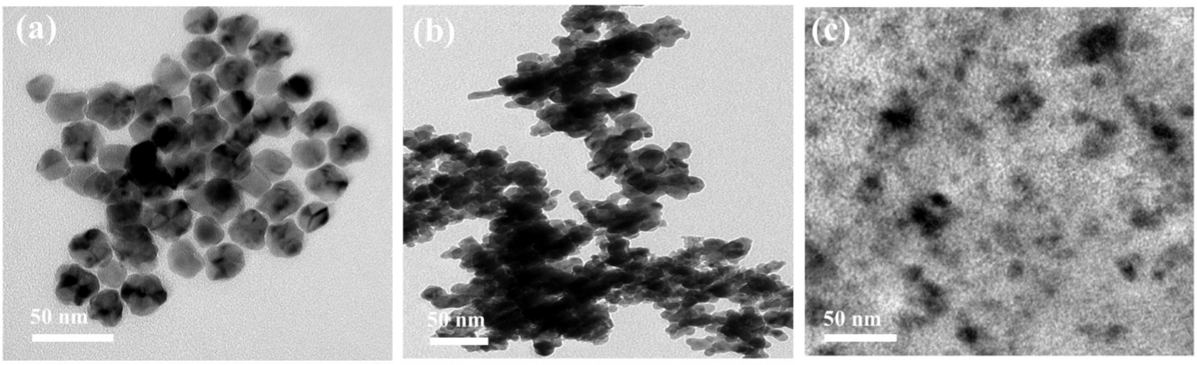


Figure S3. TEM of the catalyst after the stability test in 0.1 M $H_2SO_4$ (a) Pd nanocube, (b) Pd nanosponge, and (c) Pd/C.

Table S2. Electrochemical activity comparison of our prepared catalysts related to the previously reported works.

| **Catalyst** | **Medium/ scan rate/ Reference electrode** | **Maximum current (mA cm$^{-2}$) density/voltage (V)** | **Ref.** |
|---|---|---|---|
| Pd nanocube<br>Pd nanosponge | 0.1M $H_2SO_4$<br>50mV/s<br>Ag/AgCl | 5.92 / 0.755<br>4.0 / 0.715 | Our work |
| PtPd Nanodendrites | 1 M KOH<br>50 mV/s<br>Ag/AgCl | 5.1 / -0.15 | [1] |
| Pd/$Ti_3C_2T_x$ | 0.1 M $HClO_4$<br>50 mV/s<br>Ag/AgCl | 0.32 / ~0.9 | [2] |
| PtNi multicubes | 0.1M KOH<br>50 mV/s<br>RHE | 0.580 / 0.65 | [3] |
| Pt DEN | 0.1M $HClO_4$<br>50 mV/s<br>$Hg/Hg_2SO_4$ | 0.200 / 0.30 | [4] |
| PdAg/C | 0.5 KOH<br>20 mV/s<br>RHE | 0.944 / 0.60 | [5] |
| Polycrystalline Pd | 0.5 M $H_2SO_4$<br>20mV/s<br>RHE | 0.18 / 0.9 | [6] |
| Pd/CMK-3-R8-1500-10 | 0.5 M $H_2SO_4$<br>20 mV/s<br>RHE | ~ 0.15 / ~ 0.9 | [7] |
| PdNiO/OLC | 0.1M $H_2SO_4$<br>50mV/s<br>RHE | 1.44 / 1.16 | [8] |

| | | | |
|---|---|---|---|
| PtPd(50%)<br>nanodendrites | 0.5 M $H_2SO_4$<br>20 mV/s<br>SCE | ~3 / ~0.6 | [9] |
| PdNiO-$CeO_2$/OLC | 0.1M $H_2SO_4$<br>50mV/s<br>RHE | 2.5 / 1.1 | [8] |
| PdAu/C | 0.5 M $H_2SO_4$<br>20 mV/s<br>Ag/AgCl | 0.57 / ~ 0.9 | [10] |
| Pd-Pd(4:1)/C | 1.0 M KOH<br>50 mV/s<br>Hg/HgO | ~ 0.18 / ~ -0.1 | [11] |
| Pt/$SnO_x$ | 1 M $HClO_4$<br>20 mV/s<br>RHE | 0.870 / 0.70 | [12] |

Table S3. EIS analysis of the catalysts measured in 0.1 M $H_2SO_4$.

| **Catalyst** | $R_s$ **(Ω)** | $R_{ct}$ **(kΩ)** | **CPE (μF.s$^{(1-\alpha)}$)** | **α** |
|---|---|---|---|---|
| Pd nanocube | 96.90 ± 0.65 | 30.0 ± 1.36 | 55.25 ± 0.63 | 0.757 |
| Pd nanosponge | 99.40 ± 0.78 | 47.5 ± 2.8 | 42.00 ± 0.01 | 0.817 |
| Pd/C | 146.20 ± 0.11 | 74.3 ± 1.2 | 25.30 ± 2.20 | 0.911 |

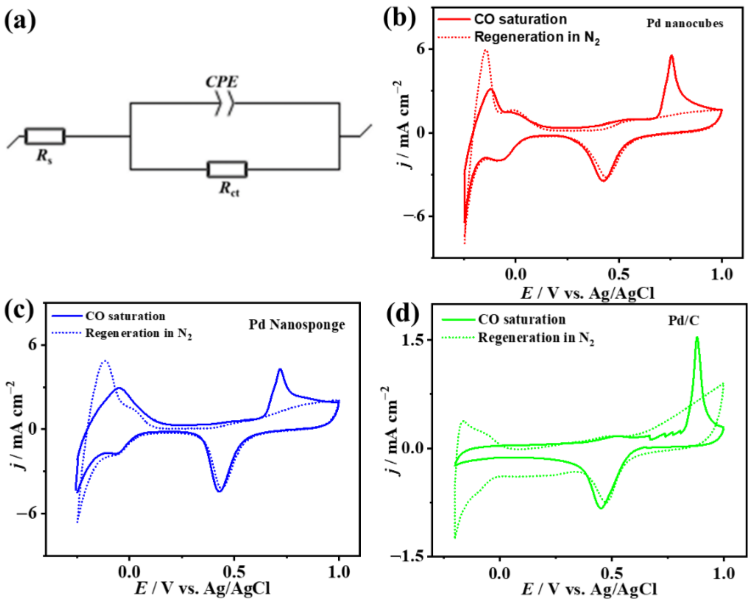


Figure S4. Voigt electrical equivalent circuit (a) and regeneration of the Pd catalysts after CO gas was switched to $N_2$ for 1 h (b-d).

Table S4. EIS analysis of the catalysts measured in 0.1 M KOH.

| Catalyst | $R_s$ (Ω) | $R_{ct}$ (kΩ) | CPE (μF.s$^{(1-\alpha)}$) | $\alpha$ |
|---|---|---|---|---|
| Pd nanocube | 96.87 ± 0.65 | 30.17 ± 1.3 | 38.25 ± 0.63 | 0.817 |
| Pd nanosponge | 93.34 ± 0.54 | 40.49 ± 2.8 | 15.82 ± 1.90 | 0.757 |
| Pd/C | 99.40 ± 0.79 | 47.49 ± 2.8 | 40.50 ± 1.30 | 0.821 |

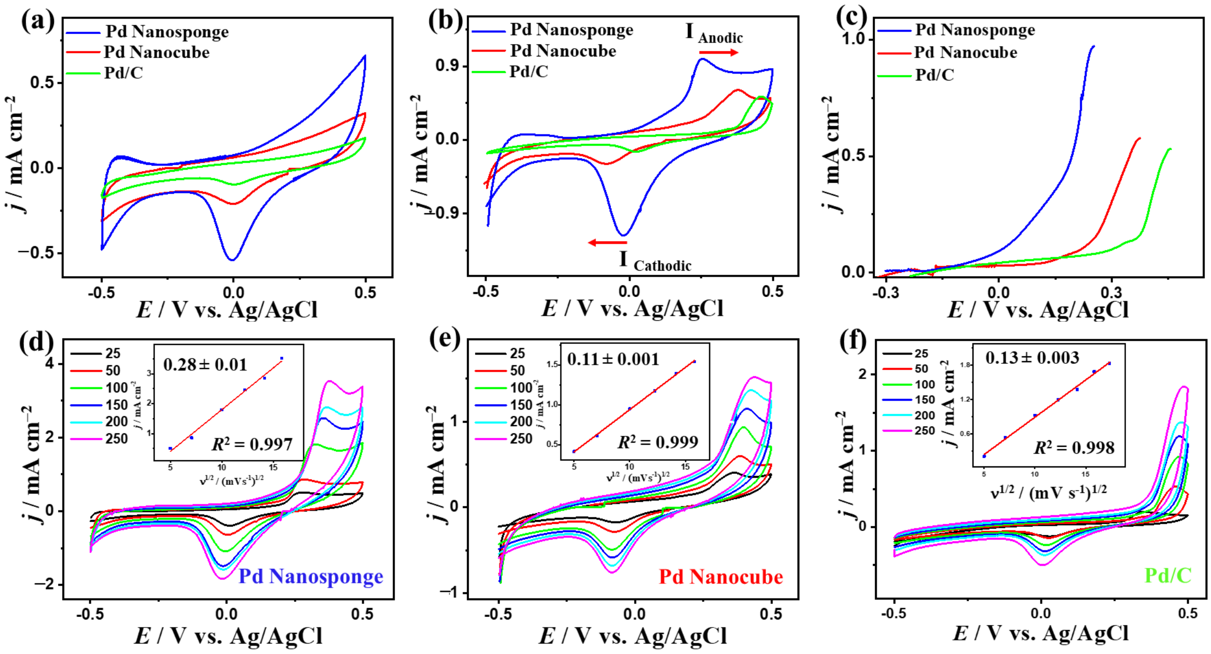


Figure S5. CV curves in $N_2$-saturated 0.5 M $NaHCO_3$ (a), CO-saturated 0.5 M $NaHCO_3$ at 50 mV/s (b), LSV at 50 mV/s (c), CV curves at different scan rates and their related plots of $I_f$ *vs.* $v^{1/2}$ of Pd nanosponge, Pd nanocube, and Pd/C (d-f), in CO-saturated 0.5 M $NaHCO_3$.

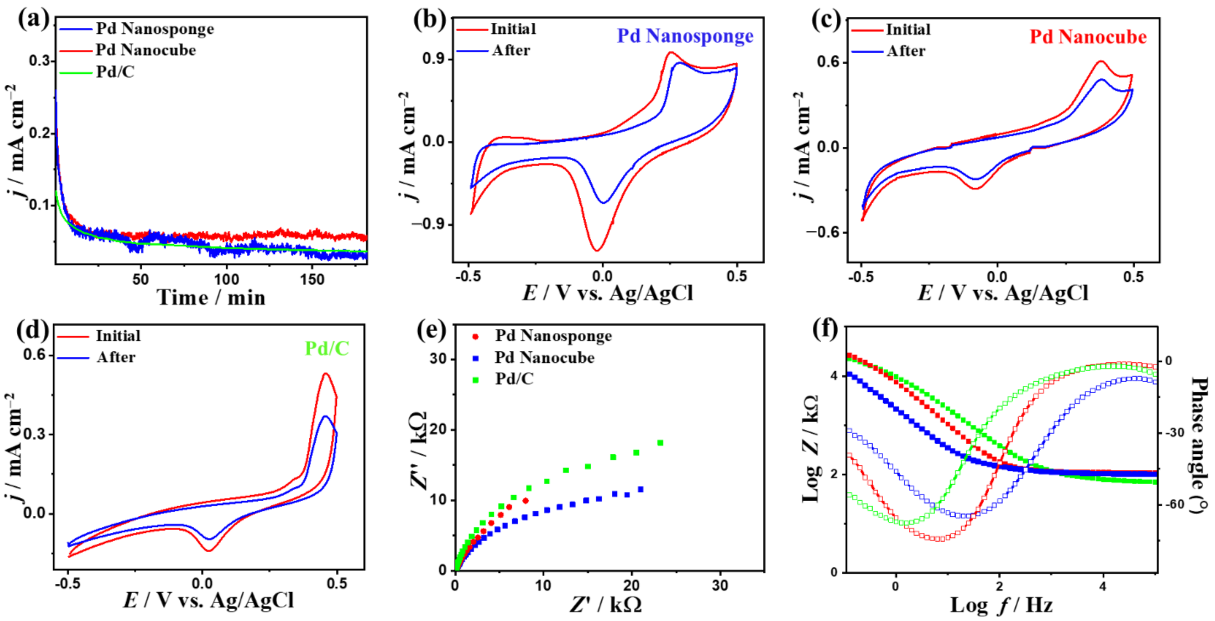


Figure S6. CA tests measured in CO-saturated 0.5 M $NaHCO_3$ (a), CV curves measured after CA (b-d), EIS (e), and Bode plots (f) of Pd nanosponge, Pd nanocube, and Pd/C.

Table S5. EIS analysis of the catalysts measured in 0.5 M $NaHCO_3$.

| Catalyst | $R_s$ (Ω) | $R_{ct}$ (Ω) | CPE (μF.s$^{(1-\alpha)}$) | α |
|---|---|---|---|---|
| Pd nanosponge | 74.34 ± 0.56 | 21.00 ± 0.746 | 82.26 ± 1.5 | 0.771 |
| Pd nanocube | 108.80 ± 0.87 | 74.86 ± 0.11 | 18.93 ± 0.35 | 0.848 |
| Pd/C | 120.1 ± 0.22 | 81.16 ± 0.01 | 16.03 ± 0.13 | 0.878 |

## References


1. Eid, K.; Ahmad, Y.H.; Yu, H.; Li, Y.; Li, X.; AlQaradawi, S.Y.; Wang, H.; Wang, L. Rational one-step synthesis of porous PtPdRu nanodendrites for ethanol oxidation reaction with a superior tolerance for CO-poisoning. *Nanoscale* **2017**, *9*, 18881-18889.
2. Salah, B.; Eid, K.; Abdelgwad, A.M.; Ibrahim, Y.; Abdullah, A.M.; Hassan, M.K.; Ozoemena, K.I. Titanium Carbide (Ti3C2Tx) MXene Ornamented with Palladium Nanoparticles for Electrochemical CO Oxidation. *Electroanalysis* **2022**, *34*, 677-683.
3. Wu, F.; Eid, K.; Abdullah, A.M.; Niu, W.; Wang, C.; Lan, Y.; Elzatahry, A.A.; Xu, G. Unveiling one-pot template-free fabrication of exquisite multidimensional PtNi multicube nanoarchitectonics for the efficient electrochemical oxidation of ethanol and methanol with a great tolerance for CO. *ACS Appl. Mater. Interfaces* **2020**, *12*, 31309-31318.
4. Weir, M.G.; Myers, V.S.; Frenkel, A.I.; Crooks, R.M. In situ X-ray absorption analysis of~ 1.8 nm dendrimer-encapsulated Pt nanoparticles during electrochemical CO oxidation. *ChemPhysChem* **2010**, *11*, 2942-2950.
5. Jurzinsky, T.; Cremers, C.; Pinkwart, K.; Tübke, J. On the influence of Ag on Pd-based electrocatalyst for methanol oxidation in alkaline media: a comparative differential electrochemical mass spectrometry study.*Electrochim. Acta* **2016**, *199*, 270-279.

6. Fang, L.-l.; Tao, Q.; Li, M.-f.; Liao, L.-w.; Chen, D.; Chen, Y.-X. Determination of the real surface area of palladium electrode. *Chin. J. Chem. Phys.* **2010**, *23*, 543-548.
7. Celorrio, V.; Sebastián, D.; Calvillo, L.; García, A.; Fermin, D.J.; Lázaro, M.J. Influence of thermal treatments on the stability of Pd nanoparticles supported on graphitised ordered mesoporous carbons. *Int. J. Hydrogen Energy* **2016**, *41*, 19570-19578.
8. Ipadeola, A.K.; Haruna, A.B.; Abdullah, A.M.; Al-Hajri, R.S.; Viter, R.; Ozoemena, K.I.; Eid, K. Technology. Ternary PdNiO nanocrystals-ornamented porous CeO 2/onion-like carbon for electrooxidation of carbon monoxide: unveiling the effect of supports and electrolytes. *Catal. Sci. Technol.* **2023**, *13*, 3035-3046.
9. Asmussen, R.M.; Adams, B.D.; Chen, S.; Shah, B.; Chen, A.J.J.o.E.C. Synthesis and electrochemical study of PtPd nanodendrites. *J. Electroanal. Chem.* **2013**, *688*, 151-157.
10. Ulas, B.; Kivrak, A.; Aktas, N.; Kivrak, H. Nanotubes; Nanostructures, C. Carbon monoxide and formic acid electrooxidation study on Au decorated Pd catalysts prepared via microwave assisted polyol method. *Fuller. Nano-tub. Carbon Nanostructures* **2019**, *27*, 545-552.
11. Wang, Y.; Nguyen, T.S.; Liu, X.; Wang, X. Novel palladium–lead (Pd–Pb/C) bimetallic catalysts for electrooxidation of ethanol in alkaline media. *J. Power Sources* **2010**, *195*, 2619-2622.
12. Matsui, T.; Fujiwara, K.; Okanishi, T.; Kikuchi, R.; Takeguchi, T.; Eguchi, K. Electrochemical oxidation of CO over tin oxide supported platinum catalysts. *J. Power Sources* **2006**, *155*, 152-156.
